# Green synchronous spectrofluorimetric analysis of remdesivir, the first approved antiviral, with levodropropizine as add-on therapy for covid-19: application in their pharmaceutical dosage form, and spiked human plasma

**DOI:** 10.1186/s13065-025-01480-8

**Published:** 2025-05-02

**Authors:** Sobhy M. El-Adl, Abdalla A. El-Shanawani, Eman A. Madbouly, Ahmed S. Abdelkhalek

**Affiliations:** Department of Medicinal Chemistry, Faculty of Pharmacy, Zagazig University, Zagazig, Egypt

**Keywords:** Remdesivir, Levodropropizine, Synchronous fluorescence, Eco-scale and GAPI greenness assessment

## Abstract

It was the first time that a spectrofluorimetric approach for the simultaneous analysis of remdesivir and levodropropizine had been achieved. This study aims to propose an accurate and sensitive second-derivative synchronous spectrofluorimetric approach for measurement of remdesivir and levodropropizine in different matrices simultaneously without the need for prior separation. The proposed approach measured the synchronous fluorescence intensity of pharmaceuticals under research at a constant wavelength difference (Δλ) = 130 nm. For the quantitative analysis of remdesivir and levodropropizine, the peak amplitudes of the second derivative were measured at 390 and 399 nm, respectively. The procedure was completely validated and demonstrated outstanding linearity in the concentration ranges of 5–150 ng mL^− 1^ and 10–600 ng mL^− 1^ for remdesivir and levodropropizine, respectively. The new method was used to quantitatively analyze both drugs in their pharmaceutical dosage form, synthetically formulated mixture and spiked human plasma. A statistical comparison of the results with other published analytical techniques revealed no significant difference. The validation of the procedure was successfully completed in compliance with ICH guidelines. In terms of greenness, EcoScale and GAPI greenness tools were used to evaluate the analytical methodology.

## Introduction

COVID-19 is considered the most catastrophic public health emergency of the 20th century. This is a viral respiratory disease that evolves quickly [1]. This disease often presents with many cases of atypical pneumonia and is caused by RNA virus known as SARS-CoV2 [2]. Before evolving into acute respiratory distress syndrome, it also exhibits a number of other symptoms, including fever, tiredness, diarrhea, and coughing [3]. Despite several vaccination approvals, they have not yet been completely effective in controlling the life-threatening pandemic [4]. The advent of virus mutations, a lack of effective alternative therapeutic techniques, and inadequate and unavailable immunization supplies are some of the possible causes of this [5]. Due to the lengthy and multi-step process involved in authorizing a novel therapy for use in humans, the quickest and easiest approach was to employ FDA-approved medications like remdesivir and favipiravir [6]. 

**Scheme 1:**
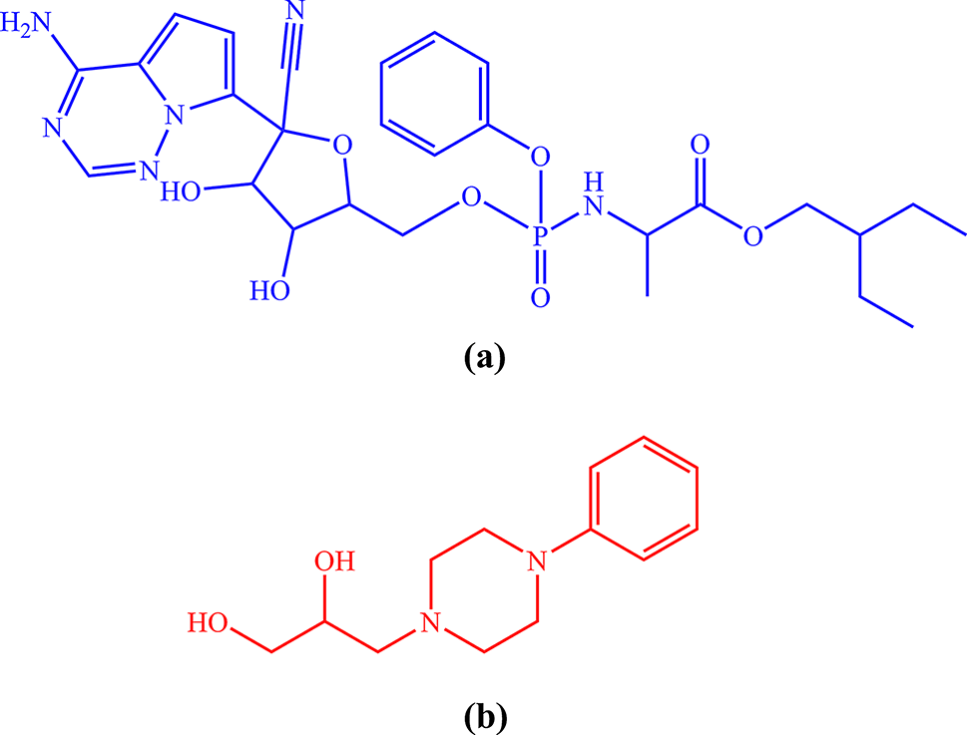
Structural formulas of remdesivir (**a**) and levodropropizine (**b**)

Remdesivir, Scheme 1a, is a broad-spectrum antiviral drug that attaches itself to the RNA polymerase of the virus and stops RNA transcription early, therefore preventing viral reproduction. Remdesivir has been investigated in numerous clinical trials as a COVID-19 therapy. FDA approved intravenous remdesivir in October 2020 for treating COVID-19 hospitalized patients [7, 8].

A variety of analytical methods for measuring remdesivir were previously puplished including electrochemical [9, 10], chromatographic [11–14], spectrofluorimetric [15–19], and spectrophotometric [20–25] approaches.

In both acute and post-acute phases of the disease, coughing is a distinctive and crucial symptom of COVID-19. Coughing not only induces discomfort in patients but also raises the possibility of respiratory droplet transmission in the general population [26]. To treat COVID-19 symptoms, antitussive medications such as levodropropizine, shown in Scheme 1b, are used. Levodropropizine, an antitussive medication with a peripheral mechanism of action, has a favorable safety and tolerability profile. Research has shown that levodropropizine exhibits comparable effectiveness and fewer sedative effects when compared to other antitussive medications, such as dihydrocodeine or dextromethorphan, which operate centrally to suppress coughing [26, 27]. levodropropizine may have a peripheral site of action and be able to alter how vagal C-fibers react to chemical stimuli, according to earlier investigations [28, 29]. 

A variety of analytical methods for measuring levodropropizine were previously puplished including electrochemical [30, 31] spectrophotometric [32, 33], spectroflourimetric [34, 35], and chromatographic [36–39] approaches.

Fluorescence spectrometry’s great sensitivity makes it a valuable analytical tool for chemical quantification. Selectivity issues may arise in multicomponent analysis due to overlapping broadband spectra. Synchronous fluorescence spectroscopy (SFS) offers advantages such as simple spectra, good selectivity, and little interference. SFS is a straightforward and efficient approach for getting quantitative data in a single measurement due to its restricted spectrum [40–42]. 

Because fluorescence spectrum overlapping problems are typically present, the use of spectrofluorimetry for quantifying multicomponents is limited. The synchronous detection mode, which scans both the emission and excitation spectra at the same time, can help to alleviate this issue. This method narrows the spectral bands, increasing band resolution and decreasing overlap. Furthermore, the resolution and selectivity of synchronous spectra can be improved by using mathematical derivatization techniques such as first- or second-order derivatives. These techniques work together to increase the clarity and accuracy of the analysis [43–47]. 

The fluorescence spectra of remdesivir and levodropropizine exhibit significant overlap when first recorded. In light of this, the authors are motivated to establish a second derivative synchronous spectrofluorimetric technique that will allow the medications to be identified specifically. The successfully devised approach was used for simultaneous determination of the co-administered drugs in different matrices.

## Experimental

### Instrumentation

A one-centimeter quartz cell and a 150-watt Xenon light were utilized in conjunction with a Jasco FP-6200 spectrofluorometer. Additionally, a Scilogex RE 100-pro rotary evaporator, a Jenway 3510 pH meter (England), and a Precisa 125 A analytical balance (Switzerland) were used.

### Chemicals

EVA Pharma (Cairo, Egypt) kindly supplied Remdesivir powder (99.15%) according to the reference method [19]. Atco Pharma (Menofia, Egypt) graciously supplied levodropropizine powder (99.70%) according to the reference method [39]. Zagazig University Hospital (Zagazig, Egypt) kindly provided plasma samples, which were kept frozen at -20 °C until assessment. Throughout the entire process, fresh distillation was used for the water, and all of the compounds were of analytical grade. Sigma-Aldrich in Germany supplied acetone, ethanol, acetonitrile, and methanol. All of the solvents used were HPLC grade. Boric acid, acetic acid (Sigma-Aldrich, Germany) and orthophosphoric acid (Prolabo, France) (each prepared as a aqueous solution of concentration 0.04 M). Aqueous solution of NaOH (0.2 M) (El-Nasr Company, Egypt) was prepared. Phosphoric acid, boric acid and acetic acid (each prepared as 0.04 M solutions) were combined to generate Britton-Robinson buffer [48]. The buffer solutions’ pH was then adjusted using the proper concentration of 0.2 M sodium hydroxide.

Remdesivir-Eva^®^ vials: acquired from the local market, this product is labeled to contain 100 mg/ 20 mL of remdesivir and is produced by EVA Pharma (Cairo, Egypt); batch number 2009561–2009563 – 2009566–2009567).

Tussistop^®^ tablet: acquired from the local market, each tablet is stated to contain 60 mg of levodropropizine. It is made by Atco Pharma (batch number 220811).

### Standard solutions

To prepare the stock solution (1 mg/mL), 100 mg of FPV and 100 mg of levodropropizine were dissolved in methanol (70 mL), sonicated for 15 min, and then the volume was completed to 100 mL with methanol. After transferring the proper aliquot into a 10 mL volumetric flask, the final standard solution was obtained. Using methanol to dilute the stock solution, a 10 µg/mL working solution was obtained.

### General analytical procedure

#### Development of calibration graphs

With a micropipette, aliquots from standard solutions of remdesivir and levodropropizine (10 µg/mL) ranging from 50 to 1500 ng for remdesivir and 100–6000 ng for levodropropizine were put into two distinct sets of 10-mL volumetric flasks. After that, the Britton-Robinson buffer was then added in an amount of 1.5 mL at pH 4. Methanol was added to the solutions to make 10 mL, and then mixed. Next, spectrofluorimetric analysis was performed on each flask’s solution using synchronous mode at Δλ = 130 nm. The experiment produced synchronous spectra, which were subsequently converted into second-order derivative spectra using data points = 7. For remdesivir and levodropropizine, the resulting peak amplitudes were recorded at respective wavelengths of 390 and 399 nm.

#### Assay of binary mixture

In 10-mL volumetric flasks, different aliquots of remdesivir and levodropropizine were transferred from their respective working standard solutions and diluted with methanol, making sure that concentrations of both drugs fell within their established linear ranges. Then, each laboratory mixture using was analyzed using the general fluorescence procedure.

#### Optimization of experimental factors

The concentrations of remdesivir and levodropropizine were kept at 120 ng/mL and 400 ng/mL, respectively, for all optimization evaluations.

##### Effect of Δλ

After repeating the general procedures under Sect. “Development of calibration graphs” at various Δλ (50–200 nm), the intensity of synchronous fluorescence of remdesivir and levodropropizine and the capacity to resolve the overlapped spectra of the two medications were explored.

##### Effect of diluent

After repeating the general procedures under Sect. “Development of calibration graphs” the synchronous fluorescence intensity values were compared using various dilution solvents, including, acetonitrile, methanol, water, ethanol and acetone.

##### Effect of pH of the buffer

After repeating the general procedures under Sect. “Development of calibration graphs” with several buffers of varying pH, a comparison was made between the synchronized fluorescence intensity levels.

##### Effect of volume of the buffer

By varying quantities of Britton-Robinson buffer (pH 4), the overall procedures under Sect. “Development of calibration graphs” were repeated, a comparison was made between the synchronized fluorescence intensity levels.

#### Assay of remdesivir and levodropropizine in their pharmaceutical dosage form (single & co-formulated)

##### Remdesivir

Four vials of Remdesivir-Eva^®^ (100 mg/ 20 mL vial) were properly mixed. Accurately, 0.2 mL, containing 1 mg of remdesivir, were put into a 100 mL volumetric flask. The flask was then filled with methanol to a volume of approximately 70 mL. After vigorously shaking the solution for 15 min, it was sonicated for 30 min. Methanol was added to the volume to reach 100 mL, to get a concentration of 10 µg/mL.

##### Levodropropizine

Ten 60 mg Tussistop^®^ tablets were pulverized coarsely and then weighed. After precisely weighing the powder that weighed the appropriate amount equal to 10 mg of levodropropizine, it was put into a 100 mL volumetric flask. The flask was then filled with methanol to a volume of approximately 70 mL. After vigorously shaking the solution for fifteen minutes, it was sonicated for thirty minutes. Methanol was added to the volume to reach 100 mL, filtered to get a concentration of 100 µg/mL, then methanol was used to dilute the solution to 10 µg/mL.

##### Co-formulation of remdesivir and levodropropizine

Remdesivir and levodropropizine fixed-dose tablets were unavailable, hence the fixed-dose combination was developed. Four Tussistop^®^ tablets (60 mg/tablet) and four Remdesivir-Eva^®^ vials (100 mg/20 mL vial) were well combined. A precise two mL of this mixture (10 mg of remdesivir and 6 mg of levodropropizine) was put into a 100 mL volumetric flask. The flask was then filled with methanol to a volume of approximately 70 mL. After vigorously shaking the solution for fifteen minutes, it was sonicated for thirty minutes. After adding methanol to fill the capacity to 100 mL, the mixture was filtered to produce a concentration of 100 µg of remdesivir and 60 µg of levodropropizine per mL. This concentration was then diluted using the same solvent to produce 10 µg of remdesivir and 6 µg of levodropropizine per mL. Utilizing aliquots that covered the range of the working concentration, the overall method was repeated for the medications under study. Based on the corresponding regression equations, the contents of pharmaceutical formulations containing remdesivir and levodropropizine were ascertained.

#### The puplished methods

- The published protocol for remdesivir analysis is synchronous spectrofluorometric method at Δλ = 130 nm. This method involves mathematical transformation to the 1st order derivative spectra, which enables the determination of remdesivir at 384 nm [19].
- The published protocol for levodropropizine is an HPLC method with an Inertsil^®^ C18 column and acetonitrile-containing mobile phase (0.1% triethylamine in water, volumetric 50:50) at pH = 3. The wavelength for UV detection was 240 nm, while 1 mL per minute was the flow rate [39].

#### Analysis of remdesivir and levodropropizine in spiked human plasma

Different aliquots from the standard solutions of remdesivir and levodropropizine (10 µg mL^− 1^) were put into 15 mL centrifuge tubes containing 1 mL of plasma sample free of drugs. Next, 3 mL of methanol was added in order to denaturize the protein. The solutions in the centrifuge tubes were vortexed for 2 min, and centrifuged for 30 min at 4000 rpm. The resulting supernatants, free of proteins, were evaporated to dryness under vacuum using a rotary evaporator. After dissolving the dry residue in methanol, it was put in 10-mL volumetric flasks and filled to the top with methanol. This procedure was repeated for each drug at various concentrations within the working range. Both remdesivir and the levodropropizine contents have been estimated by corresponding regression analysis.

### Greenness of the analytical methods estimation

Since 1999, there has been much discussion on the principles of green analytical chemistry (GAC), which emphasize using cleaner solvents, lowering energy usage, and using fewer reagents [49]. Tools like the Green Analytical Procedure Index (GAPI) and the Eco-Scale penalty points were used to assess how environmentally friendly the proposed approach was [49, 50]. However, because they only cover a small number of assessment criteria and consider them as non-continuous functions, these tools have drawbacks. None of these metric systems assess analytical methods using all 12 GAC principles, and their findings might not offer a thorough summary of the analytical methodology or in-depth details on the structure of risks. Thus, to evaluate the method’s greenness, AGREE, an extra tool, was also employed [51]. The developed approach was rigorously validated to assure compliance with green chemistry principles.

## Results & discussion

The current work proposed a straightforward and sensitive second derivative synchronous spectrofluorimetric method for the concurrent measurement of remdesivir and levodropropizine in different matrices without the need for prior separation.

### Spectral characteristics

**Fig. 1:**
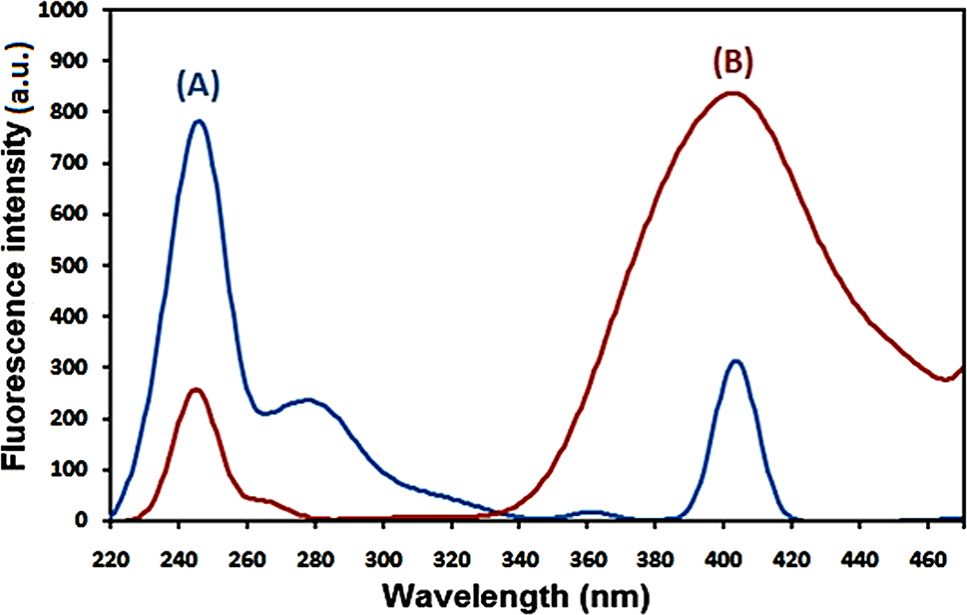
Excitation (**A**) and emission (**B**) spectra of remdesivir in methanol

**Fig. 2:**
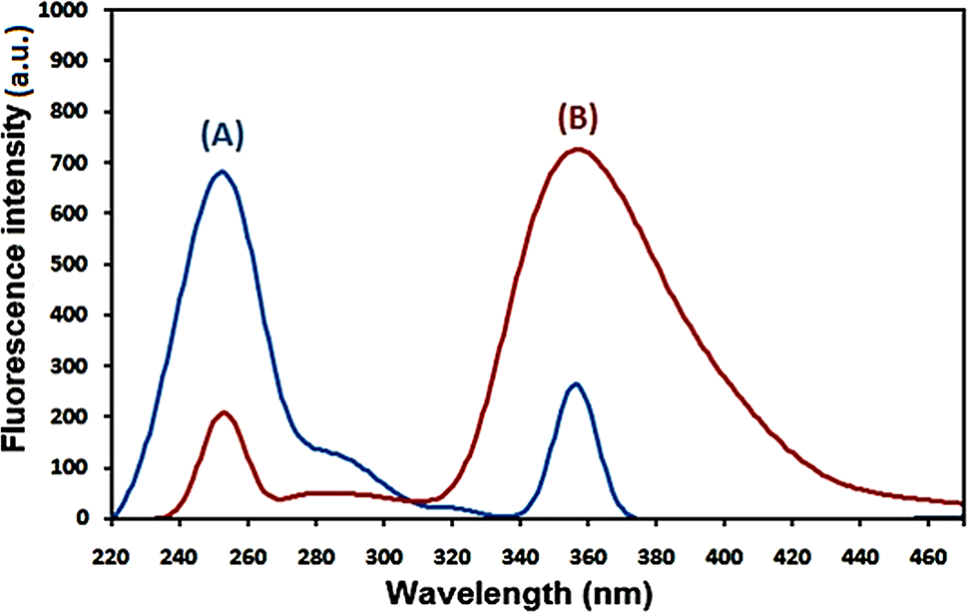
Excitation (**A**) and emission (**B**) spectra of levodropropizine in methanol

**Fig. 3:**
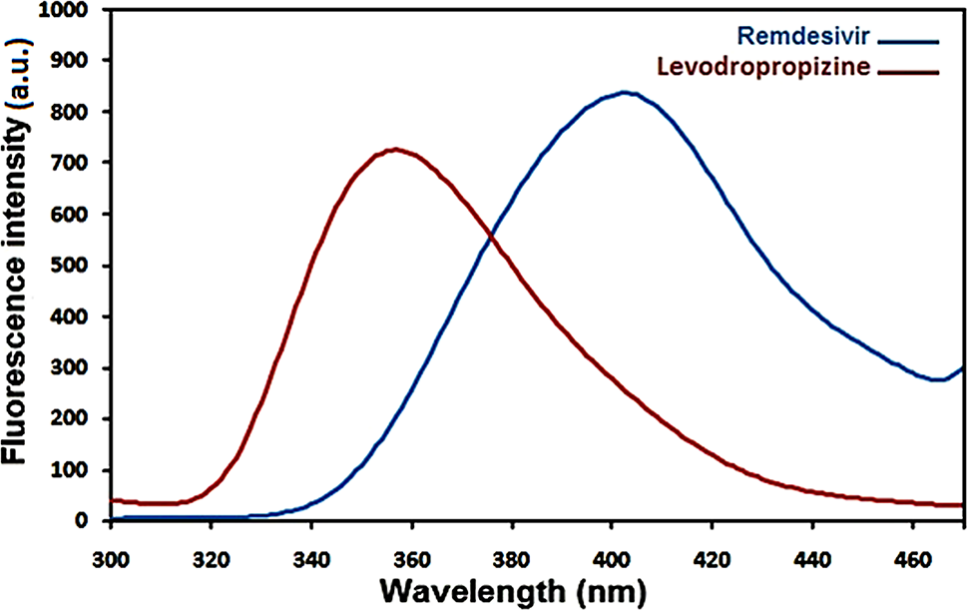
Overlain emission spectra of remdesivir and levodropropizine in methanol after excitation at 250 nm

**Fig. 4:**
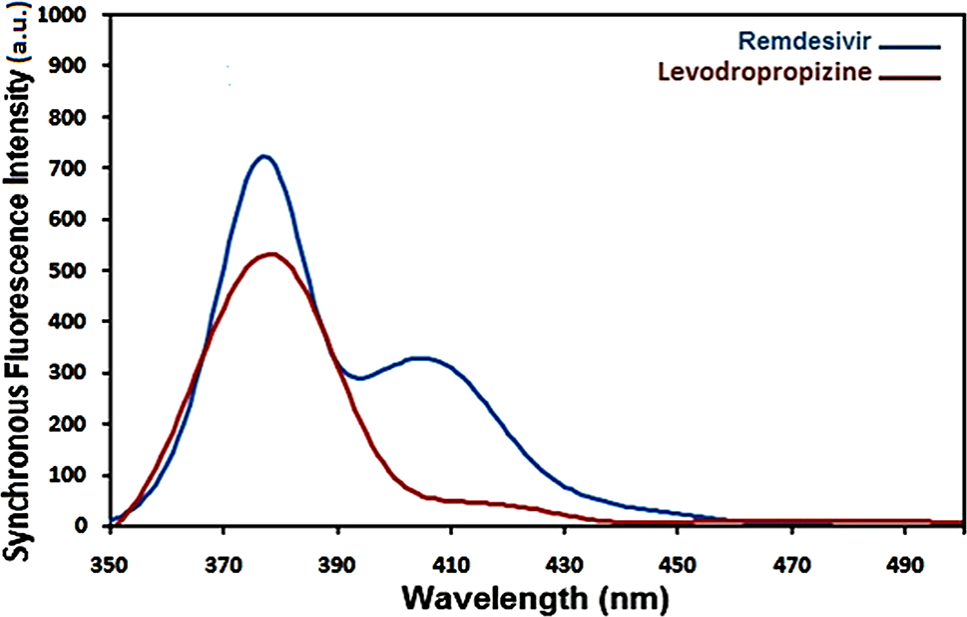
Synchronous fluorescence spectra of remdesivir and levodropropizine using Δλ = 130 nm

**Fig. 5:**
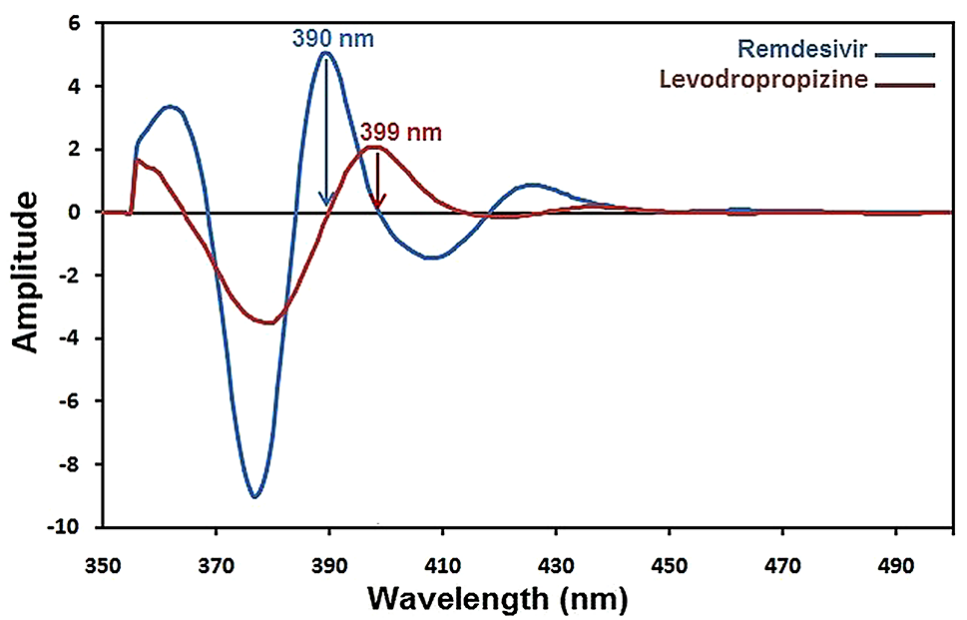
Second derivative synchronous fluorescence spectra of remdesivir and levodropropizine using Δλ = 130 nm

Native fluorescence is present in remdesivir and levodropropizine and the excitation and emission spectra of remdesivir in methanol are displayed in Figs. 1 and 2. Remdesivir emission may be observed at 403 nm (λex. = 246 nm), while levodropropizine emission may be seen at 357 nm (λex. = 252 nm). Figure 3 illustrates the overlapped emission spectra of remdesivir and levodropropizine, which made it difficult to determine the two compounds by standard fluorescence. Additionally, the issue remains unresolved even after derivatizing standard fluorescence spectra. In order to resolve this overlap, one must measure the synchronous fluorescence at Δλ = 130 nm in order to determine the second derivative (the 1st derivative is inappropriate). As illustrated in Figs. 4 and 5, the resulting second derivative synchronous fluorescence spectra of remdesivir and levodropropizine enabled their respective measurements at 390 and 399 nm.

### Experimental conditions optimization

The impact of diverse experimental *conditions* on the fluorescence intensities of the aforementioned drugs was investigated and optimized. Every variable was assessed separately, with no changes made to the others. These variables included the diluting solvent type, buffer volume, pH, and Δλ selection.

In terms of resolution, sensitivity, and characteristics, the ideal Δλ value is crucial when utilizing the synchronous fluorescence scanning approach. Band width, signal strength, and spectral form can all be directly impacted. The separation was best achieved at Δλ of 130 because it produced two separate peaks with well-formed outlines and lessened the spectral interference that each compound caused.

**Fig. 6:**
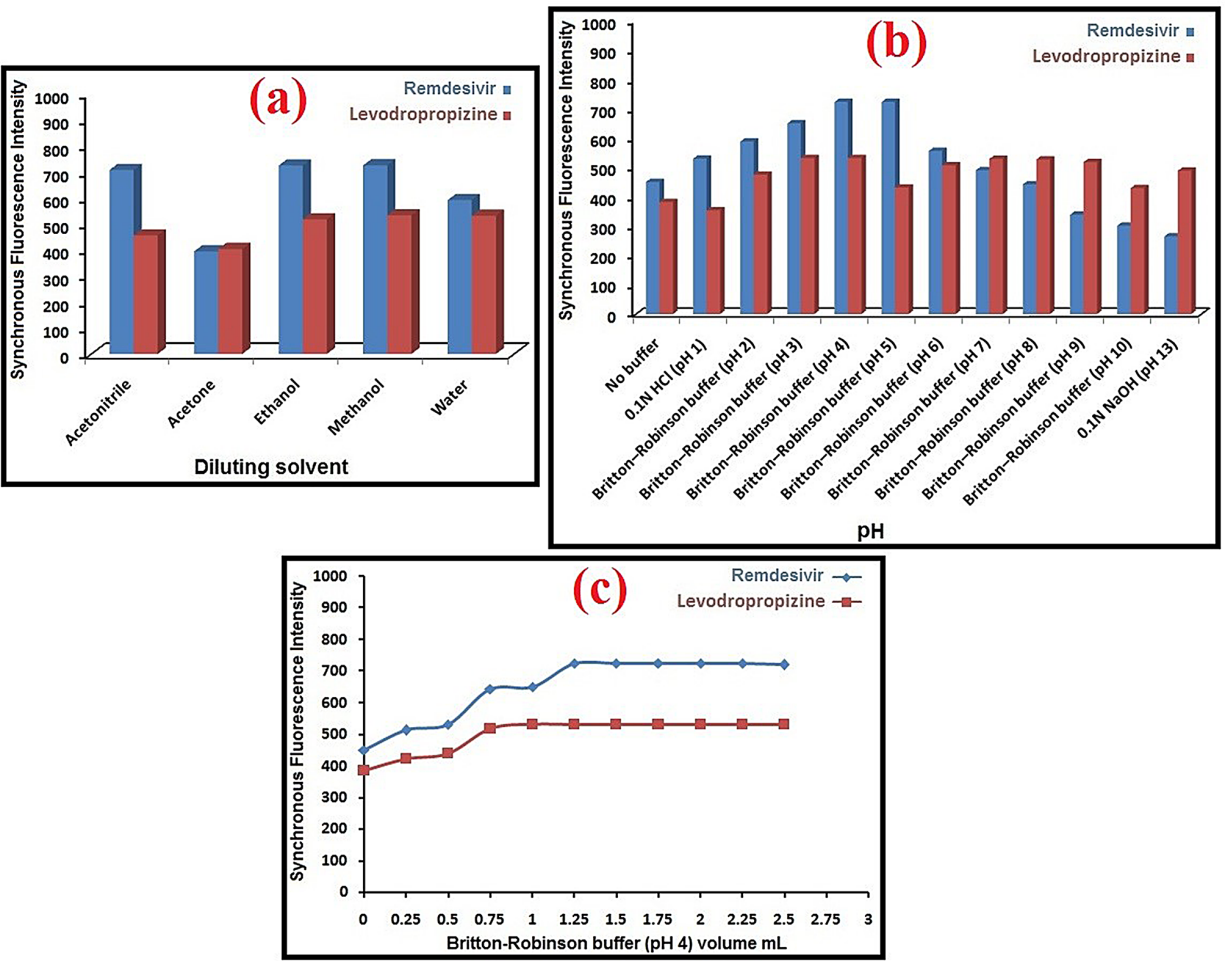
Optimization of experimental conditions for remdesivir and levodropropizine with regard to diluting solvent (**a**), buffer type (**b**), and buffer volume (**c**)

As seen in Fig. 6, methanol was determined to be the ideal diluting solvent, and the ideal synchronous fluorescence intensity was attained when pH was adjusted using of Britton–Robinson buffer (1.5 mL) at pH = 4.

### Method validation

The suggested techniques were successfully validated and produced satisfactory results in accordance with ICH criteria [52, 53]. 

#### Range and linearity

The amplitudes of the second derivative of the synchronous spectra at 390 nm for remdesivir and 399 nm for levodropropizine were plotted against the corresponding drug concentrations in µg/mL to construct the calibration graph for each drug under the given experimental conditions. For remdesivir and levodropropizine, the regression curve was shown to be linear between 5 and 150 ng/mL and 10 and 600 ng/mL, respectively. Table 1 displayed the regression data. The calibration graphs’ strong linearity was demonstrated by the coefficient of determination value.

**Table 1:** Analytical performance data for the proposed method

Parameters	Remdesivir	Levodropropizine
Wavelength (nm)	390	399
Linearity range (ng/mL)	5 ─ 150	10 ─ 600
Slope ± SD	0.0340 ± 0.0006	0.0039 ± 0.0001
Intercept ± SD	0.9964 ± 0.0199	0.4763 ± 0.0095
LOD (ng/mL)	1.415	3.021
LOQ (ng/mL)	4.287	9.156
Coefficient of determination (r^2^)	0.9996	0.9995
Accuracy (R%) ^**a**^	99.03	98.94
**Precision (RSD%)** ^**b**^		
**-** Repeatability **-** Intermediate precision	1.290 1.151	0.870 1.341
**Robustness (R**^**c**^**%**± **RSD %)**		
**-** Δλ (± 1 nm) **-** pH (± 0.1) **-** Britton-Robinson buffer volume (± 0.1 mL)	99.87 ± 1.102 98.43 ± 0.497 100.72 ± 0.691	100.24 ± 1.351 99.07 ± 1.093 99.61 ± 1.572
Molecular weight	602.6 g/mol	236.31 g/mol
Solubility	Soluble in Ethanol and methanol	Soluble in Ethanol, DMSO, and dimethyl formamide (DMF)
pKa (Strongest Acidic)	10.23	14
pKa (Strongest Basic)	0.65	7.65

^**a**^ Nine determinations on average (3 concentrations repeated three times)

^**b**^ RSD% of nine measurements (three concentrations, each repeated three times)

^**c**^ Average of three measurements

#### Limits of detection and quantification

Table 1 shows the LOQ and LOD values. The obtained values showed that the recommended method can precisely measure the drugs in spiked plasma; as a result, it may be applied in upcoming in vivo studies.

#### Accuracy and precision

The specified analytical procedures was used in triplicate to determine three concentration levels covering the linearity range of the drugs under study (50, 100, and 120 ng/mL for remdesivir and 50, 200, and 400 ng/mL for remdesivir). This allowed for the calculation of the method’s accuracy, which was measured as the mean percent recovery (R%).

The procedure described for the triplicate determination of three concentration levels covering the linearity range of the studied drugs (50, 100, and 120 ng/mL for remdesivir and 50, 200, and 400 ng/mL for levodropropizine) within one day for repeatability and on three consecutive days for intermediate precision was used to calculate the precision of the method, which was expressed as the percent relative standard deviation (RSD%).

The acquired good R%, as mentioned in Table 1, demonstrated the accuracy of the suggested procedure. Table 1 illustrates that the method’s great precision was demonstrated by the tiny values of RSD%.

#### Specificity

Synthetic mixes with varying proportions of the medications under study were made and thoroughly mixed. The combinations were then examined utilizing the method’s previously outlined basic approach (Sect. “Development of calibration graphs”). Excellent, satisfactory outcomes were attained and are shown in Table 2. The suggested technique was used to determine the dosage forms of remdesivir and levodropropizine when they were combined in the lab, as shown in Table 3. To assess the impact of the matrix on the identification of the two medications, specificity was also assessed by using the conventional addition approach to pharmaceutical preparations that had already been analysed. Table 4 of the data showed that the suggested method was able to evaluate the medicine selectively and free from excipient interference.

**Table 2:** Assay of laboratory prepared mixture of remdesivir and levodropropizine by the suggested flourimetric approach

Laboratory prepared mixture(ng/mL)		Recovery%^*^	
Remdesivir	Levodropropizine	Remdesivir	Levodropropizine
25	15	101.74	99.13
50	30	100.48	99.90
75	45	100.60	98.47
100	60	101.59	100.94
125	75	101.62	99.97
100	100	101.02	98.52
**Mean ± RSD%**		101.17 ± 0.551	99.49 ± 0.964

^*^ Average of 3 analyses

**Table 3:** Assay of Remdesivir and Levodropropizine using the suggested flourimetric approach in single-component dosage forms and co-formulated dosage forms

Remdesivir-Eva^®^ 100 mg/vial		Tussistop^®^ 60 mg/tablet		Co-formulated dosage form			
				Remdesivir		Levodropropizine	
Conc. (ng/mL)	*R*% *	Conc. (ng/mL)	*R*% *	Conc. (ng/mL)	*R* %*	Conc. (ng/mL)	*R* % *
25	99.38	15	98.27	25	98.94	15	98.12
50	98.71	30	99.01	50	98.62	30	99.20
75	101.95	45	98.12	75	101.84	45	98.34
100	100.69	60	100.39	100	100.56	60	100.47
125	101.27	75	101.46	125	101.37	75	100.86
**Mean**	100.40	**Mean**	99.45	**Mean**	100.27	**Mean**	99.40
**RSD%**	1.331	**RSD%**	1.447	**RSD%**	1.434	**RSD%**	1.241

^*^ Average of 3 analyses

**Table 4:** Recovery analysis of Remdesivir and Levodropropizine by employing the recommended flourimetric approach and standard addition technique

Pharmaceutical (ng/mL)	Remdesivir		Levodropropizine	
	Pure added (ng/mL)	R%*	Pure added (ng/mL)	R%*
Remdesivir 50 (49.31) ^*^ Levodropropizine 30 (29.76) ^*^	30	98.23	20	101.95
	50	98.3	30	101.73
	60	100.38	100	99.41
**Mean**		98.97		101.03
**RSD%**		1.235		1.394

^*^ Average of 3 analyses

#### Robustness

The robustness of the proposed method was assessed by following the procedure outlined in Sect. “Development of calibration graphs”, with minor modifications to the optimal Britton-Robinson buffer volume (± 0.1 mL), pH (± 0.1), and Δλ (± 1 nm). Only a single parameter was altered in each instance, keeping all other factors unchanged. As indicated in Table 1, the procedure’s robustness was confirmed by the RSD % of the responses being less than 2%, indicating that the slight alterations specified had no major impact on the synchronous fluorescence intensity.

### Greenness of the analytical methods Estimation

Using multiple greenness evaluation tools yields more accurate results and allows for a more comprehensive comparison of different analytical method [54]. Three greenness assessment metrics, the analytical eco-scale, and the green analytical procedure index (GAPI) [49, 50], were applied to assess the proposed spectroflourimetric method. Furthermore, a comparison between the reference methods and the specified method’s greenness was conducted. The analytical eco-scale is a semi-quantitative approach for estimating the impact of analytical methodologies on the environment. The penalty points for the analytical procedure are determined based on two main parameters. First among the parameters is the reagent parameter, which is determined by assessing the reagents’ amounts, effects on the environment, physical risks, and health concerns. The second parameter pertains to instrumentation, taking into account factors such as energy usage, work dangers, and waste generation. To calculate the greenness evaluation score, deduct the penalty points assigned to each criteria from 100. The green approach is classified as excellent or acceptable, or inadequate green based on its total score value. In Table 5, the calculated analytical eco-score of the described spectroflourimetric method (89 score) versus the reference method (88 score) for remdesivir and (86 score) for levodropropizine indicating that our method is as excellent green as reference method with also slight superiority.

The GAPI metrics use five pictograms and a unique symbol to assess the environmental friendliness of each step in the analytical process. It uses a visual graph with a chromaticity scale of red, yellow, and green to methodically classify each phase’s level of greenness [49, 50]. Table 5 displays the GAPI pictogram for the suggested process, which had eight green zones, four yellow zones, and three red zone. As a result, our proposed spectroflourimetric method has more green zones, making it preferable to the compared reference methods.

The third approach, the analytical greenness measure (AGREE), was shown as a 12-piece pie chart with a value in the middle. Since 1 is the optimum value, a high value around 1 and a more green-colored shape using a red-yellow-green color scale indicate how green an analytical technique is. The analysis’s final score of 0.69 indicates how ecologically friendly the method was in comparison to all reference methods as in Table 5 [51].

**Table 5:**
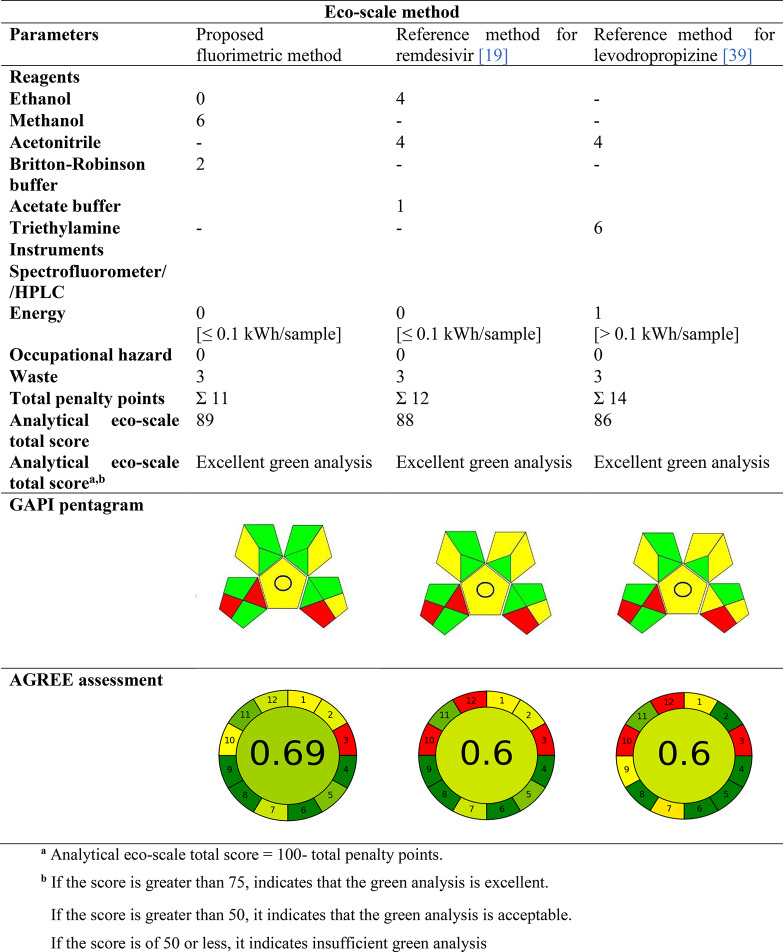
Greenness estimation of the developed and reference methods

## Pharmaceutical applications

As displayed in Table 3, remdesivir and levodropropizine were ascertained as single dosage forms or as co-formulated dosage forms in the laboratory as part of the suggested approach. As can be seen in Table 4, the findings of the usual addition technique proved that additives and excipients had no effect. Statistics were used to compare the results with those attained by the previously mentioned methods [19, 39]. There were no significant differences observed using the student’s t-test and F-test at the 95% confidence level [55], showing that the proposed method for analyzing the tested substance in its pharmaceutical dose form is accurate and precise, as indicated in Table 6.

**Table 6:** Statistical comparison between the results obtained by the proposed and reference methods

Parameters	Remdesivir		Levodropropizine	
	Proposed method	Reference method [19]	Proposed method	Reference method [39]
Number of measurements	5	5	5	5
Mean Recovery %	100.40	98.93	99.45	101.14
RSD %	1.331	0.740	1.447	0.930
Variance	1.785	0.535	2.070	0.885
Student’s *t*-test^**a**^ (2.306)	2.158	——	2.201	——
*F*-value^**a**^ (6.388)	3.334	——	2.339	——

^**a**^ The values in parenthesis are tabulated values of “*t* ” and “*F* ” at (P = 0.05)

## Human plasma application

The standard spectroflourimetric methods were used to measure both medications in human plasma in order to demonstrate their superior sensitivity. Both remdesivir and levodropropizine had plasma C_maxes_ that were higher than their respective LODs, ranging from 57.5 to 4420 ng/mL and 261.22 to 325.46 ng/L, respectively [56, 57]. Table 7 displays the satisfactory results obtained for the quantitative analysis of remdesivir and levodropropizine in spiked human plasma. As a result, the suggested spectroflourimetric methods can be applied to the pharmacokinetic investigation of remdesivir and levodropropizine without interference from the plasma matrix.

**Table 7:** Application of the suggested flourimetric approach for the Estimation of Remdesivir and Levodropropizine in spiked human plasma

Remdesivir			Levodropropizine		
Added (ng/mL)	Found* (ng/mL)	Recovery%	Added (ng/mL)	Found* (ng/mL)	Recovery%
15	14.58	97.20	15	14.22	94.80
20	19.17	95.85	20	18.57	92.85
50	48.93	97.86	30	27.91	93.03
100	94.87	94.87	60	57.06	95.10
125	118.92	95.14	75	70.35	93.80
**Mean ± RSD%**		96.18 ± 1.353	**Mean ± RSD%**		93.92 ± 1.080

* Average of five analyses

## Conclusion

This study developed and verified a highly sensitive second derivative synchronous spectrofluorimetric technique to determine remdesivir and levodropropizine simultaneously. The approach proved to be successful in analyzing both medications in pharmaceutical dosage forms without interference from excipients, making it suitable for quality control laboratories. The approach is effective for determining drugs in spiked human plasma due to its high sensitivity and low interference from the plasma matrix. When compared to other instruments like gas chromatography and HPLC, the used instrument is simple and reasonably priced. The recommended method uses methanol as the solvent because it is more readily available, less expensive, and environmentally friendly. Using green solvents has advantages for the environment, making it a competitive solution for quality control.

## Data Availability

The datasets used and/or analyzed during the current study are available from the corresponding author on reasonable request.

## Abbreviations

ICH: International Conference on Harmonization
GAPI: Green Analytical Procedure Index
